# Genetic Evidence for an Indispensable Role of Somatic Embryogenesis Receptor Kinases in Brassinosteroid Signaling

**DOI:** 10.1371/journal.pgen.1002452

**Published:** 2012-01-12

**Authors:** Xiaoping Gou, Hongju Yin, Kai He, Junbo Du, Jing Yi, Shengbao Xu, Honghui Lin, Steven D. Clouse, Jia Li

**Affiliations:** 1 School of Life Sciences, Lanzhou University, Lanzhou, China; 2 Department of Botany and Microbiology, University of Oklahoma, Norman, Oklahoma, United States of America; 3 School of Life Sciences, Sichuan University, Chengdu, China; 4 Department of Horticultural Science, North Carolina State University, Raleigh, North Carolina, United States of America; University of Michigan, United States of America

## Abstract

The *Arabidopsis thaliana* Somatic Embryogenesis Receptor Kinases (SERKs) consist of five members, SERK1 to SERK5, of the leucine-rich repeat receptor-like kinase subfamily II (LRR-RLK II). SERK3 was named BRI1-Associated Receptor Kinase 1 (BAK1) due to its direct interaction with the brassinosteroid (BR) receptor BRI1 *in vivo*, while SERK4 has also been designated as BAK1-Like 1 (BKK1) for its functionally redundant role with BAK1. Here we provide genetic and biochemical evidence to demonstrate that SERKs are absolutely required for early steps in BR signaling. Overexpression of four of the five *SERKs*—*SERK1*, *SERK2*, *SERK3/BAK1*, and *SERK4/BKK1*—suppressed the phenotypes of an intermediate *BRI1* mutant, *bri1-5*. Overexpression of the kinase-dead versions of these four genes in the *bri1-5* background, on the other hand, resulted in typical dominant negative phenotypes, resembling those of null *BRI1* mutants. We isolated and generated single, double, triple, and quadruple mutants and analyzed their phenotypes in detail. While the quadruple mutant is embryo-lethal, the *serk1 bak1 bkk1* triple null mutant exhibits an extreme de-etiolated phenotype similar to a null *bri1* mutant. While overexpression of *BRI1* can drastically increase hypocotyl growth of wild-type plants, overexpression of *BRI1* does not alter hypocotyl growth of the *serk1 bak1 bkk1* triple mutant. Biochemical analysis indicated that the phosphorylation level of BRI1 in *serk1 bak1 bkk1* is incapable of sensing exogenously applied BR. As a result, the unphosphorylated level of BES1 has lost its sensitivity to the BR treatment in the triple mutant, indicating that the BR signaling pathway has been completely abolished in the triple mutant. These data clearly demonstrate that SERKs are essential to the early events of BR signaling.

## Author Summary

Brassinosteroids (BRs) are a group of plant hormones critical for plant growth and development. BRs are perceived by a cell-surface receptor complex including two distinctive receptor kinases, BRI1 and BAK1. Whereas BRI1 is a true BR-binding receptor, BAK1 does not appear to have BR-binding activity. Therefore, BAK1 is likely a co-receptor in BR signal transduction. The genetic significance of BAK1 was not clearly demonstrated in previous studies largely due to functional redundancy of BAK1 and its closely related homologues. It was not clear whether BAK1 plays an essential role or only an enhancing role in BR signaling. In this study, we identified all possible BAK1 redundant genes in the *Arabidopsis thaliana* genome and generated single, double, triple, and quadruple mutants. Detailed analysis indicated that, without BAK1 and its functionally redundant proteins, BR signaling is completely disrupted, largely because BRI1 has lost its ability to activate downstream components. These studies provide the first piece of loss-of-functional genetic evidence that BAK1 is indispensable to the early events of the BR signaling pathway.

## Introduction

Brassinosteroids (BRs) are naturally produced plant hormones regulating many developmental processes from seed germination to flowering and senescence [1]. BR deficiency or response mutants show typical phenotypic defects including decreased rate of seed germination, rounded and epinastic rosette leaves, extremely dwarfed stature, delayed flowering time, reduced male fertility, postponed leaf senescence, and extremely de-etiolated phenotypes grown under dark conditions [2]–[5]. Although both plants and animals use steroids as growth regulators, the signaling pathways in the two kingdoms are divergent [6]. While animal steroids are known to be perceived by nuclear receptors which can directly regulate gene transcription upon ligand binding, BRs are sensed by a single-pass transmembrane leucine-rich repeat receptor-like protein kinase (LRR-RLK) named Brassinosteroid Insensitive 1 (BRI1) and two BRI1 paralogs, BRI1-Like 1 (BRL1) and BRL3 [3], [7]–[9]. When BR is absent, BRI1 was found to exist as a homodimer whose cytoplasmic domain interacts with a membrane-anchored protein termed BRI1 Kinase Inhibitor 1 (BKI1), blocking the interaction between the kinase domains of BRI1 and its co-receptor BRI1-Associated Receptor Kinase 1 (BAK1) [10]–[13]. Recent crystal structure analyses demonstrated that the LRRs of BRI1 form an extremely twisted helical solenoid structure and the hydrophobic pocket formed by LRRs and the “island” domain provides a direct binding site for BRs [14], [15]. The ligand-receptor interaction initiates the BR signaling cascade, mostly via reversible phosphorylation and dephosphorylation [16].

It was proposed that during early events of BR signaling, the BR receptor BRI1 and its co-receptor BAK1 follow a reciprocal and sequential phosphorylation process before downstream components can be activated [17]. Interaction of BR with the extracellular domain of BRI1 triggers a conformational change of the cytoplasmic domain of BRI1, causing phosphorylation of BKI1 on a conserved tyrosine residue, resulting in the dissociation of phosphorylated BKI1 from BRI1 [12], [18]. It is likely that the BRI1 kinase domain is autophosphorylated and activated via an intermolecular mechanism, and the activated BRI1 then recruits BAK1, via a kinase-to-kinase and extracellular domain-to-extracellular domain double lock mechanism, in close proximity and phosphorylates several Thr residues within the activation loop of BAK1, activating the co-receptor [19], [20]. The active BAK1 then phosphorylates multiple residues within the juxtamembrane and carboxyl terminus regions of BRI1, fully activating BRI1 and creating proper docking sites for association of other BRI1 downstream components such as BR-Signaling Kinases (BSKs) [21]. The activated BSKs can inhibit kinase activity of a negative regulator Brassinosteroid Insensitive 2 (BIN2) by activating a protein phosphatase named *bri1* Suppressor 1 (BSU1) to dephosphorylate a phosphotyrosine residue (pTyr200) on BIN2 [22]. Inactivation of BIN2 causes the accumulation of two unphosphorylated transcription factors Brassinazole-Resistant 1 (BZR1) and *bri1*-Ems-Suppressor 1 (BES1) in nuclei, directly mediating the expression of BR responsive genes [23]–[28]. Phosphorylated forms of BES1 and BZR1, on the other hand, are trapped in cytoplasm by interacting with 14-3-3 proteins and eventually degraded via a 26S proteasome-mediated pathway [29]–[32].

The role of BAK1 has been mainly defined by various gain-of-function genetic and biochemical analyses [10], [11], [17], [20]. The significance of the function of BAK1, however, has so far not been demonstrated by a loss-of-function genetic analysis which is considered as the most reliable approach to reveal biological functions of a given gene. A *BAK1* null mutant only exhibits a subtle *bri1*-like defective phenotype suggesting either additional homologues of BAK1 play redundant roles with BAK1, or BAK1 and its homologues only provide an enhancing but not an essential role to BR signaling. The significance of BAK1 and its homologues in mediating BR signaling should be determined in a mutant plant with lesions of *BAK1* and all its functionally redundant genes. Recent studies indicated that BAK1 and its homologues also play important roles in regulating several BR-independent signaling pathways such as anther development, cell-death control, and disease resistance [33]. For example, the *serk1 serk2* double mutant shows an anther defective phenotype [34], [35]; the *bak1 bkk1* double null mutant displays light-dependent spontaneous cell death at the seedling stage [36], [37]; and the *bak1* single mutant exhibits uncontrolled cell death and reduced innate immunity responses to a variety of pathogens [38]–[42]. These findings add additional complexity to our efforts towards understanding the significance of BAK1 in BR signaling pathway.

In this study, we show that four out of five members of the SERK subfamily (SERK1 to SERK4), in the wild-type *Arabidopsis* Columbia background (Col-0), may play functionally redundant roles in BR signaling. In Col-0, SERK5 contains a mutation in an important amino acid residue which likely abolishes the kinase activity of SERK5 [36]. We subsequently isolated null mutants for all four kinase active *SERKs*, and generated double, triple, and quadruple mutants using two sets of independent null mutants. Our detailed analysis indicates that dark grown *serk1 bak1 bkk1* triple mutants show a typical de-etiolated phenotype resembling a null *bri1* mutant. Physiological and biochemical analyses indicate that the triple mutant is insensitive to BR treatment similar to a null *bri1* mutant. Furthermore, the phosphorylation level of BRI1 in the triple mutant is completely unresponsive to BR treatment, suggesting that BRI1 cannot initiate BR signaling without BAK1 and its homologues. These results provide clear genetic and biochemical evidence that BAK1 plays an essential role in the BR signal transduction pathway.

## Results

### *SERK1*, *SERK2*, *BAK1*, and *BKK1* are the only members in the LRR-RLK II subfamily which can suppress *bri1-5* when overexpressed

**Figure 1:**
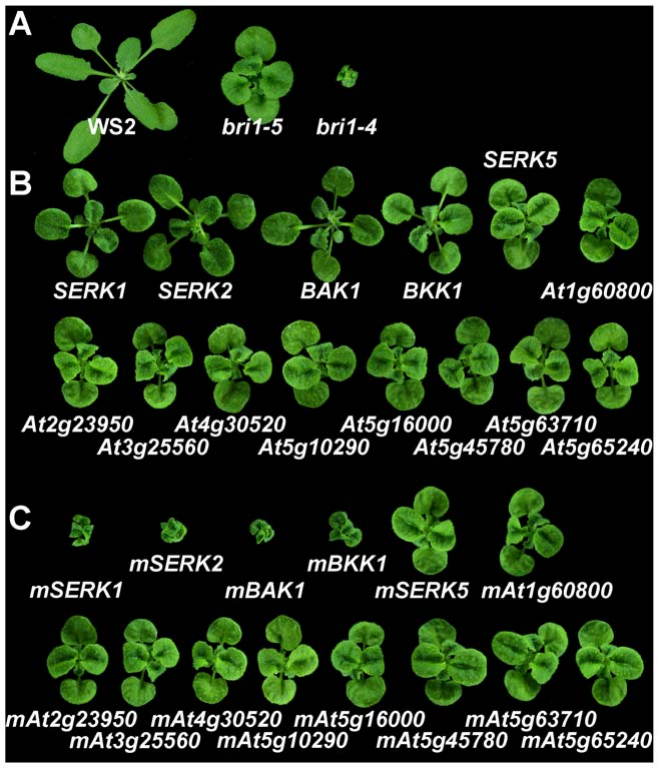
*SERK1*, *SERK2*, *BAK1*, *BKK1* are the only four genes in the LRR-RLK II subfamily playing redundant roles in mediating BR signal transduction. A. Phenotypes of wild-type *Arabidopsis* (WS2), an intermediate *BRI1* mutant, *bri1-5*, and a null *BRI1* mutant, *bri1-4*. B. Overexpression of *SERK1*, *SERK2*, *BAK1*, and *BKK1* can partially suppress the defective phenotypes of *bri1-5*. Overexpression of *SERK5* and other members in LRR-RLK II subfamily can not suppress the *bri1-5* phenotypes. C. Overexpression of kinase-inactive mutants, *mSERK1* (K330E), *mSERK2* (K333E), *mBAK1* (K317E), and *mBKK1* (K322E) dramatically enhances *bri1-5* defective phenotypes. Overexpression of *mSERK5* (K303E) and other kinase-inactive mutants in LRR-RLK II subfamily does not enhance the *bri1-5* phenotypes. Representative 18-day-old plants were photographed.

BAK1 was previously identified as a coreceptor of BRI1 in mediating BR signaling [10], [11]. Genetic data demonstrating BAK1 is essential to BR signal transduction is still lacking. If *BAK1* plays a role which is as critical as *BRI1*, a mutant plant with loss-of-function mutation of *BAK1* and all its functionally redundant genes should exhibit a typical *bri1* null mutant phenotype (Figure 1A). A *bak1* null mutant, however, shows a subtle *bri1*-like phenotype suggesting that there may be the LRR-RLK II subfamily genes that are functionally redundant with BAK1. The five SERKs are grouped in a single clade of the LRR-RLK II subfamily according to phylogenetic analyses [43], [44]. Based on sequence similarity, it is logical to hypothesize that any BAK1 functionally redundant proteins would be members of the LRR-RLK II subfamily. To test this hypothesis, all 14 LRR-RLK II members were overexpressed in the intermediate *bri1-5* mutant in the WS2 background [45]. Our results indicated that only *SERK1*, *SERK2*, *BAK1*, and *BKK1* could partially suppress *bri1-5* phenotypes when overexpressed (Figure 1B). These overexpressed transgenic plants showed elongated petioles and wild-type like bolting and flowering time.

Previous studies already indicated *in vivo* physical interactions of BRI1 with SERK1, BAK1, and BKK1, respectively [10], [11], [36], [46]. In this study, we tested the *in vivo* interaction between BRI1 and SERK2 using transgenic plants overexpressing *BRI1-FLAG* and *SERK2-GFP*. Our result indicated that BRI1-FLAG can weakly interact with SERK2-GFP *in vivo*. Unlike the interactions of BRI1 with BAK1 and BKK1, the interaction between BRI1 and SERK2 cannot be significantly enhanced with exogenously applied BR (Figure S1), but the phosphorylation of SERK2 can be dramatically enhanced by the supplementation of BR (Figure S1). These results suggest that SERK2 may have fewer roles in BR signaling compared to the other three SERKs, but unnatural manipulation such as overexpression of *SERK2* and exogenous application of BR can reveal its functions in BR signaling.

To further confirm the function of *SERKs* in BR signaling, kinase-inactive versions of all 14 LRR-RLK II members were generated by mutating a conserved lysine residue in the ATP binding site of each of these genes. These mutated genes were named *mSERK1*, *mSERK2*, *mBAK1*, *mBKK1*, etc. All 14 mutated genes were overexpressed in *bri1-5*. If mutated mSERKs still can interact with bri1-5, the resulting transgenic plants should show a dominant negative phenotype. Our results indicated that most of the transgenic plants obtained for all the four constructs of *mSERKs* showed phenotypes resembling that of a null *bri1* mutant, such as *bri1-4* [45] (Figure 1C). These data suggest that *SERK1*, *SERK2*, *BAK1*, and *BKK1* are the only 4 *LRR-RLK II* genes that are involved in BR signaling.

### *serk1 bak1 bkk1* triple null mutant shows null *bri1*-like phenotypes in the dark

**Figure 2:**
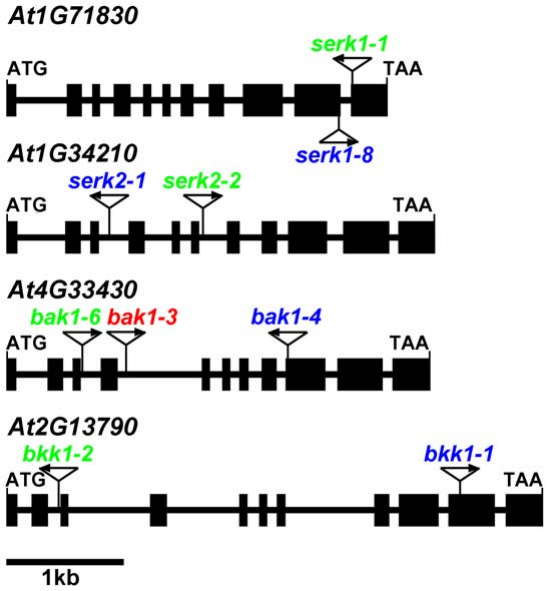
Two independent sets of T-DNA insertion null mutants used in this study. Exons are indicated with filled black boxes. Lines between boxes represent introns. The insertion sites are shown with triangles and the T-DNA orientations are indicated with arrows. The T-DNA insertion mutants labeled with blue were the 1^st^ set of single null mutants used to create double, triple and quadruple mutants. The ones labeled with green were the 2^nd^ set of mutants used to generate double, triple, and quadruple mutants.

To better understand the genetic significance of SERKs in BR signaling, single, double, triple, and quadruple mutants for all four *SERKs* were generated. Two independent sets of T-DNA insertion lines for all the four *SERKs* were obtained from the *Arabidopsis* Biological Resource Center (ABRC) (Figure 2). RT-PCR reactions were performed to confirm that all the lines used do not express full-length wild-type mRNAs (Figure S2). Therefore, these lines are likely null mutants. A novel *BRI1* T-DNA insertion line named *bri1-701* was also obtained from ABRC. *bri1-701* not only showed no full-length mRNA expression but also exhibited a phenotype identical to a typical null *bri1* mutant. *bri1-701* was therefore used as a null *bri1* control throughout the entire studies. Double *serk* mutants were generated by crossing two different *serk* mutants. Because *serk1 serk2*, *serk1 bak1*, and *bak1 bkk1* showed male sterility, reduced male fertility, and cell death phenotypes respectively, we had to be strategic about generating triple mutants, as discussed in materials and methods.

**Figure 3:**
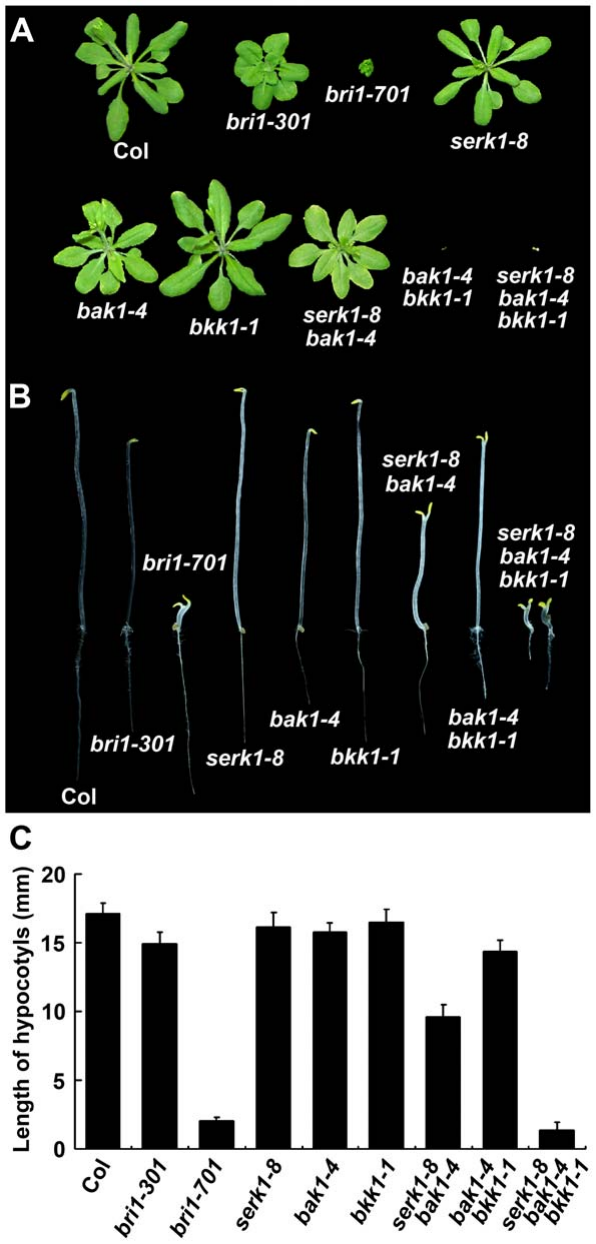
Representative loss-of-function mutant phenotypes of *SERKs*. A. Representative loss-of-function phenotypes of 20-day-old *SERK* mutants in the light. Only *bak1-4* shows a subtle *bri1*-like phenotype among the single knock-out mutants. The double knock-out mutant *serk1-8 bak1-4* shows phenotypes similar to the *bri1* weak allele, *bri1-301*; and *bak1-4 bkk1-1* shows a seedling-lethality phenotype at the early developmental stage [36]. The triple knock-out mutant *serk1-8 bak1-4 bkk1-1* shows phenotypes similar to the *bak1-4 bkk1-1* mutant plants. B. Representative loss-of-function phenotypes of *SERK* mutants grown in the dark for 5 days. The mutant *bri1-701*, a T-DNA insertion mutant of *BRI1*, shows a typical null *bri1*-like phenotype in the dark with opened cotyledons, short and swollen hypocotyls. The double knock-out mutant *serk1-8 bak1-4* shows partially de-etiolated phenotypes with opened cotyledons and semi-dwarfed hypocotyls. The triple knock-out mutant *serk1-8 bak1-4 bkk1-1* shows a de-etiolated phenotype almost identical to that of the *BRI1* null mutant, *bri1-701*. C. Measurements of the dark-grown seedlings shown in B. Error bars represent standard deviation (SD).

We first phenotypically analyzed the first set of single mutants of the four *SERKs*, *serk1-8*, *serk2-1*, *bak1-4*, and *bkk1-1* (Figure 2). Only *bak1-4* showed subtle *bri1*-like phenotypes such as shortened petioles, reduced rosette size, and reduced height when grown in the light. When grown in the dark, *bak1-4* showed slightly shortened hypocotyls compared to wild-type seedlings as reported previously (Figure 3; [10], [11]). None of the other *SERK* mutants showed any visible defective phenotypes. We then generated double mutants with the four single mutants. Among all 6 possible double mutants generated, only *serk1-8 bak1-4* showed a weak *bri1-*like phenotype which is more severe than the *bak1-4* single mutant, including more compact rosette leaves and more shortened hypocotyls (Figure 3). As a matter of fact, the hypocotyls of *serk1-8 bak1-4* are only about half of the length of wild-type (Figure 3B, 3C), which is still significantly taller than the *bri1-701* null mutant, suggesting that BR signaling is severely but not completely disrupted in *serk1-8 bak1-4*. We confirmed that *serk1-8 serk2-1* shows a defective male gametogenesis phenotype; and *bak1-4 bkk1-1* shows seedling lethality phenotypes about two weeks after germination as previously reported [34]–[36].

Our priority in these genetic studies was to investigate whether knocking-out *BAK1* and its functionally redundant genes in a single plant can completely abolish BR signaling pathway and recapitulate a *bri1*-like phenotype. Because *bak1-4 bkk1-1* shows a seedling lethality phenotype due to failure of a light-dependent cell death control [36], we did not anticipate observing a light-grown null *bri1*-like phenotype for the triple or quadruple mutants. Therefore, the phenotypes of the triple and quadruple mutants were mainly analyzed within the dark-grown conditions. To our surprise, some of the *serk1-8 serk2-1 bak1-4* triple mutant displayed an embryo-defective phenotype, and the *serk1-8 serk2-1 bak1-4 bkk1-1* quadruple mutant are most likely embryo-lethal (data not shown). Our attention was drawn to one of the triple mutants, *serk1-8 bak1-4 bkk1-1*, because this mutant showed an extreme de-etiolated phenotype similar to that of *bri1-701* including shortened hypocotyls and opened cotyledons (Figure 3B, 3C).

To further confirm that the triple mutant phenotypes result from the null mutations of the corresponding *SERKs*, we generated double, triple, and quadruple mutants using a different set of single null mutants, *serk1-1*, *serk2-2*, *bak1-6*, and *bkk1-2* (Figure 2). All the defective phenotypes observed are similar to the mutants generated by the first set of null alleles (Figure S3). Most importantly, *serk1-1 bak1-6 bkk1-2* also showed a severe *bri1*-like de-etiolated phenotype (Figure S3). The phenotypic resemblance suggests that the BR signaling pathway is abolished in the *serk* triple mutant similar to that in the *bri1* null mutant.

### The phenotypes of *SERK* mutants can be complemented by corresponding *SERK* genes

**Figure 4:**
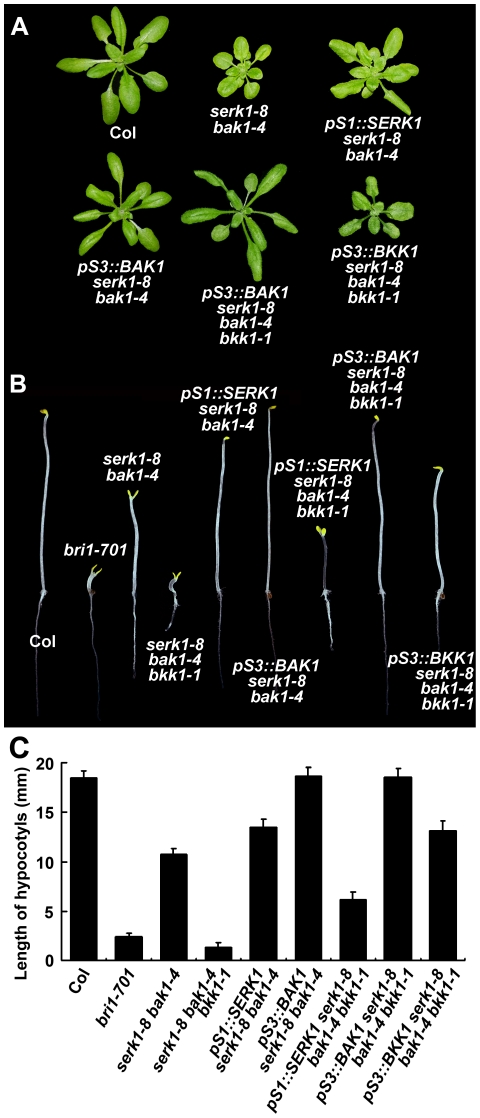
The phenotypes of *SERK* mutants can be complemented by each of the *SERK*s. A. Rescued mutant phenotypes of 21-day-old plants in the light. The *pS1::SERK1-GFP* construct restored the *serk1-8 bak1-4* mutant phenotype to a typical *bak1-4* like mutant phenotype, and the *pS3::BAK1-GFP* construct restored the *serk1-8 bak1-4* mutant phenotype to a wild-type like plant. The *pS3::BAK1-GFP* and *pS3::BKK1-GFP* constructs restored the *serk1-8 bak1-4 bkk1-1* mutant phenotype to almost a wild-type like plant. However, *pS1::SERK1-GFP* can not rescue the *serk1-8 bak1-4 bkk1-1* phenotype in the light. B. Rescued mutant phenotypes of 5-day-old seedlings in the dark. The *pS1::SERK1-GFP* and *pS3::BAK1-GFP* constructs restored the *serk1-8 bak1-4* mutant phenotypes. The *pS1::SERK1-GFP* construct partially restored the *serk1-8 bak1-4 bkk1-1* mutant phenotypes to the *bak1-4 bkk1-1* double mutant phenotypes with opened cotyledons and taller hypocotyls than the triple mutant. The *pS3::BAK1-GFP* and *pS3::BKK1-GFP* constructs restored the *serk1-8 bak1-4 bkk1-1* mutant phenotype to a wild-type like seedling. C. Measurements of the dark-grown seedlings shown in B. Error bars represent SD.

To verify that the observed phenotypes are caused by the loss-of-function of the *SERK* genes, we performed complementation experiments using native *SERK* promoters. Because the double, triple, and quadruple mutants generated from 2 sets of independent mutants look very similar, we performed the complementation and all other biochemical experiments using the mutants generated from the 1^st^ set of single null mutant. We were never able to clone the BKK1 promoter into these constructs; therefore, the *BAK1* promoter was used to drive the expression of *BKK1*. When native promoter-driven *SERK1* and *BAK1* were transformed into *serk1-8 bak1-4*, the double mutant phenotypes were largely rescued (Figure 4A). When *BAK1* promoter driven *BAK1* and *BKK1* were transformed into *serk1-8 bak1-4 bkk1-1* triple mutant background, the lethality phenotype of the triple mutant was also rescued (Figure 4A). When *SERK1* promoter driven *SERK1* was introduced into the *serk1-8 bak1-4 bkk1-1* background, no rescued plants were ever obtained because of cell death resulted from knock-out of *BAK1* and *BKK1* (data not shown). The transgenic plants were also grown in the dark to examine whether they restored the de-etiolated phenotype of the mutant plants. Indeed, *SERK1* and *BAK1* restored the mutant phenotypes of *serk1-8 bak1-4* in the dark. *BAK1* and *BKK1* completely rescued the mutant phenotypes of *serk1-8 bak1-4 bkk1-1* in the dark. The complementation of *SERK1* driven by its own promoter only partially restored the mutant phenotypes, producing semi-dwarf hypocotyls (Figure 4B, 4C). The reason why *pS1::SERK1* only partially rescued the hypocotyl phenotype of *serk1-8 bak1-4 bkk1-1* is not known. Results from two sets of independent null mutants and from the complementation experiments clearly indicated that the defective mutant phenotypes were caused by the null mutations in the *SERKs*.

### The *serk1 bak1 bkk1* triple null mutant is insensitive to BR treatment

**Figure 5:**
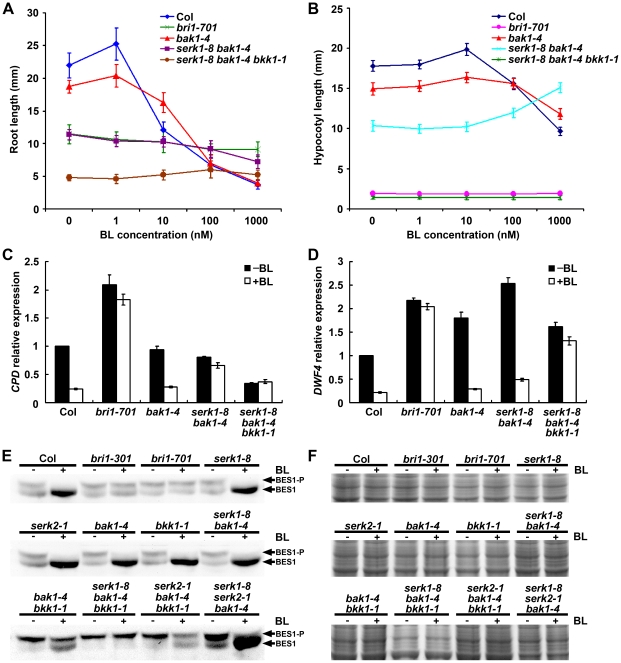
*serk1-8 bak1-4 bkk1-1* triple null mutant is insensitive to exogenous BR treatment. A, B. Root and hypocotyl growth analyses of wild-type and mutant plant seedlings grown on medium containing different 24-epiBL concentrations. A. Seven-day-old seedlings grown in the light for root growth analysis. B. Five-day-old seedlings grown in the dark for hypocotyl growth analysis. The double mutant *serk1-8 bak1-4* shows reduced sensitivity to BR treatment and the triple mutant *serk1-8 bak1-4 bkk1-1* is completely insensitive to BR treatment. Error bars represent SD. C, D. Expression of *CPD* and *DWF4* in *serk1-8 bak1-4 bkk1-1* shows responses to exogenously applied BR similarly to the null *bri1*-701 mutant but differently to wild-type seedlings. Seven-day-old seedlings were treated with or without 1 µM 24-epiBL. Relative expression level of *CPD* and *DWF4* was measured by quantitative RT-PCR. The feedback inhibition of *CPD* in *serk1-8 bak1-4* is also reduced. *ACTIN2* was used as the reference gene. The used primers are listed in Table S1. Error bars represent SD (n = 3). E, F. The phosphorylation level of BES1 is not responsive to BR treatment in the *serk1-8 bak1-4 bkk1-1* triple null mutant. E. Nine-day-old seedlings of wild-type and mutants grown in the light were treated with 0 or 1 µM 24-epiBL for 4 h. Total proteins were analyzed by an immuo-blotting assay with a specific anti-BES1 antibody. F. Coomassie blue staining showing equally loaded proteins between each pair of samples. −, without 24-epiBL treatment; +, with 24-epiBL treatment. BES1-P, phosphorylated BES1; BES1, unphosphorylated BES1.

To examine whether the null *bri1*-like phenotype seen in the dark grown *serk1 bak1 bkk1* triple null mutant was caused by the disruption of BR signaling, a classic root growth inhibition assay for BR sensitivity was performed [2]. If *SERKs* play an essential role in BR signaling, *SERK* mutants should show a reduced response to exogenously applied BR. Plants were grown for 7 days on half strength MS medium agar plates supplied with or without different concentrations of BR. Wild-type and *bak1-4* are sensitive to the root growth inhibition of different concentrations of 24-epiBL ranging from 1 to 1000 nM, with *bak1* showing slightly reduced sensitivity (Figure 5A). The root growth of *serk1-8 bak1-4* was insensitive to 24-epiBL from 1 to 100 nM, but showed some sensitivity at 1000 nM. Analyses of hypocotyls also showed reduced sensitivity of *serk1-8 bak1-4* to exogenously applied 24-epiBL (Figure 5B). The root and hypocotyl growth of *bri1-701* and *serk1-8 bak1-4 bkk1-1*, however, was completely insensitive to 24-epiBL at concentrations from 1 to 1000 nM. These results indicate that the triple mutant plants are insensitive to exogenously applied BR (Figure 5A, 5B). To confirm that the de-etiolated phenotype seen in the triple mutant is caused by the disruption of the BR signaling pathway instead of the general photomorphogenesis pathways, we used two constitutive photomorphogenesis (*COP1*) mutants, *cop1-4* and *cop1-6*, as controls to determine whether they also show insensitivity to exogenous BR treatment (Figure S4A, S4B). Our results indicated that although *COP1* mutants exhibit a de-etiolated phenotype, they are sensitive to exogenous BR treatment.

In Col-0, the expression of *CPD* is down-regulated by exogenously applied BR via a negative feedback mechanism. When BR signaling is disrupted as in *bri1* null mutants, however, the expression of *CPD* is not responsive to BR treatment [47]. To further confirm that the BR signaling is blocked in *serk1 bak1 bkk1* triple mutant, quantitative RT-PCR experiments were performed to detect the expression levels of *CPD* and *DWF4* in wild-type and mutant plants treated with or without BR (Figure 5C, 5D). Similar to previous reports, the expression level of *CPD* was decreased to about 20% of wild-type plants when treated with BR. In *bri1-701* null mutant, the expression of *CPD* was not significantly down-regulated when treated with BR (Figure 5C). Consistent with its weak *bri1*-like phenotype, the expression level of *CPD* in *bak1-4* was decreased dramatically similar to wild-type upon BR treatment. However, the double knock-out mutant *serk1-8 bak1-4* showed drastically reduced sensitivity to BR treatment with slightly decreased *CPD* expression upon BR treatment. The expression level of *CPD* in the BR treated *serk1-8 bak1-4 bkk1-1* triple null mutant was not decreased (Figure 5C). Similar to the *CPD* response, the expression level of *DWF4* was down-regulated to about 20% in wild-type plants when BR was applied exogenously. However, BR treated *serk1-8 bak1-4 bkk1-1* mutant showed dramatically reduced sensitivity to exogenous BR application similar to the *bri1-701* mutant plant (Figure 5D). These data indicate that the BR signaling pathway in the triple mutant of *SERKs* is blocked to the same extent as *bri1* null mutants.

### Phosphorylation level of BES1 is not responsive to BR in the *serk1 bak1 bkk1* triple null mutant

To biochemically test whether the *serk1 bak1 bkk1* triple null mutant is insensitive to exogenously applied BR, the phosphorylation status of BES1 was investigated with a specific anti-BES1 antibody after the wild-type and triple mutant were treated with or without 1 µM 24-epiBL (Figure 5E, 5F; Figure S5) [48]. In Col-0 wild-type seedlings without BR treatment, two BES1 bands with almost equal signal intensity were observed, indicating that both phosphorylated and unphosphorylated BES1 were present. Upon BR treatment, unphosphorylated BES1 in wild-type was increased dramatically and phosphorylated BES1 disappeared, suggesting that the BR signaling pathway was activated. In the untreated seedlings of the weak allele of *bri1-301*, the amount of phosphorylated BES1 was slightly higher than that of unphosphorylated BES1. In the BR-treated *bri1-301* seedlings, the amount of unphosphorylated BES1 became greater than phosphorylated BES1. However, the untreated null mutant *bri1-701* showed higher levels of phosphorylated than unphosphorylated BES1, and the ratio between the two types of BES1 was not changed when treated with BR. All single *SERK* null mutants showed response to BR in a way similar to that of wild-type plants. Although *serk1-8 bak1-4* showed an enhanced *bri1*-like phenotype compared to *bak1-4* single mutant (Figure 3), BES1 phosphorylation levels in the double mutant still show a wild-type like response (Figure 5E). A previous report showed that BAK1 and BKK1 function redundantly in BR signaling [36], which is also supported by the result from this study that untreated *bak1-4 bkk1-1* seedlings showed predominantly phosphorylated BES1 protein, suggesting that BR signaling is dramatically impaired in the double mutant. Upon BR treatment, both phosphorylated and unphosphorylated BES1 were detected although the phosphorylated BES1 was still dominant, indicating that the BR signaling pathway was not disrupted completely in *bak1-4 bkk1-1*. Interestingly, the *serk1-8 bak1-4 bkk1-1* triple null mutant only showed the phosphorylated BES1 band with or without the exogenous BR application, suggesting that BR signal transduction was entirely blocked in the triple mutant. *serk2-1 bak1-4 bkk1-1* exhibited a BES1 phosphorylation response similar to *bak1-4 bkk1-1*. When *serk1-8 serk2-1 bak1-4* was treated with BR, the amount of unphosphorylated BES1 increased dramatically (Figure 5E). These results indicate that *serk2* has an undetectable effect on BR signaling in *Arabidopsis* seedlings; and BR signaling appears to be entirely blocked in the *serk1 bak1 bkk1* triple null mutant. Similar results were obtained from the second set of knockouts (Figure S5). Although BR insensitive mutants showed a de-etiolated phenotype, de-etiolated mutants do not always show BR insensitivity. For example, *cop1-4* and *cop1-6* showed wild-type like BES1 response to BR treatment (Figure S4C).

### Overexpression of *BRI1* cannot rescue the de-etiolated phenotype of the *serk1 bak1 bkk1* triple mutant, and the phosphorylation level of BRI1 in the triple mutant is unresponsive to BR treatment

**Figure 6:**
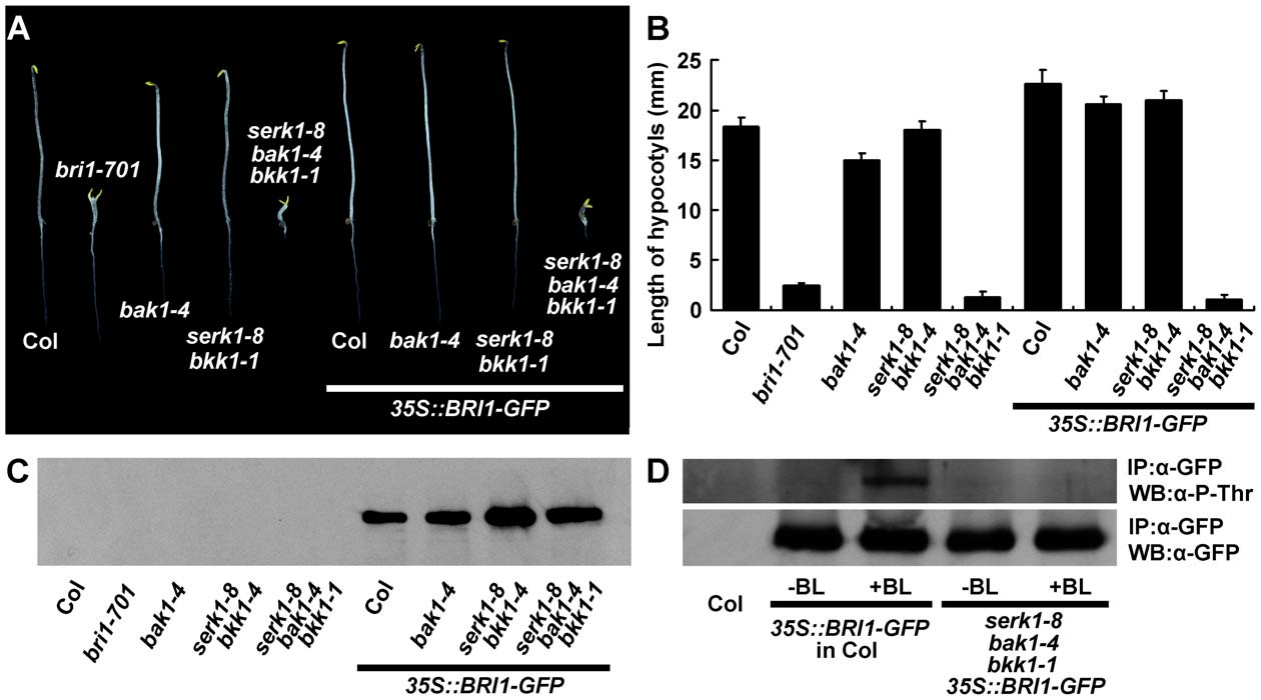
Overexpression of *BRI1* dramatically increases hypocotyl growth of wild type but does not promote hypocotyl growth of *serk1-8 bak1-4 bkk1-1*. A. *35S::BRI1-GFP* can not suppress the mutant phenotype of *serk1-8 bak1-4 bkk1-1*. Five-day-old dark grown seedlings were photographed. B. Measurements of the dark-grown seedlings shown in A. Error bars represent SD. C. Western hybridization indicates the expressed BRI-GFP fusion protein in transgenic plants. D. Phosphorylation of BRI1 does not respond to exogenously applied BR in the mutant *serk1-8 bak1-4 bkk1-1*. The membrane proteins were extracted from seven-day-old light-grown seedlings and immunoprecipitated with an anti-GFP antibody. The threonine phosphorylation levels of BRI1 were detected with a phosphoThr antibody by immuno-blotting analysis. The immunoprecipitated BRI1-GFP was immuno-blotted with an anti-GFP antibody to show equal loading. −BL, samples not treated with 24-epiBL; +BL, samples treated with 100 nM 24-epiBL for 90 min.

It was previously reported that overexpression of *BRI1* can drastically increase hypocotyl growth of wild-type plants [7], [20]. To test whether BRI1 is still able to promote hypocotyl growth of the *serk1 bak1 bkk1* triple null mutant, we overexpressed *BRI1-GFP* in wild-type and the *serk1-8 bak1-4 bkk1-1* respectively. Our results indicated that overexpression of *BRI1-GFP* can increase hypocotyl growth of wild-type plants but has no effect on the triple null mutants (Figure 6A, 6B, 6C). To understand why BRI1 has lost its physiological roles in the triple mutant, we next analyzed whether the phosphorylation levels of BRI1 can respond to exogenously applied BR. In the wild-type plants, exogenous BR treatment could significantly increase the phosphorylation levels of BRI1 as reported (Figure 6D; [17], [20]); whereas in the *serk1 bak1 bkk1* triple null mutant, phosphorylation of BRI1 remains at an extremely low level regardless of BR treatment (Figure 6D). These results suggest that BR signaling is blocked in the triple mutant most likely because BRI1 has lost its responsiveness to internal bioactive BRs.

## Discussion

The function of BAK1 in mediating BR signaling was independently identified by using a yeast-two hybrid screen for BRI1 interacting proteins and activation tagging for genetic suppressors of an intermediate *BRI1* mutant, *bri1-5* [10], [11]. Since then, numerous data supported the biochemical roles of BAK1 in regulating early events of BR signal transduction [17], [20], [49]. The biological significance of BAK1 in BR signaling, however, has never been convincingly substantiated due to lack of loss-of-function genetic evidence. If BAK1 is a key component in BR signaling, a plant lacking *BAK1* and all its functionally redundant genes should exhibit a phenotype identical to a *bri1* null mutant. In addition, the mutant plant should show no response to BR treatment in a way similar to what has been revealed for null *bri1* mutants. To resolve these questions, we set to identify all genes in the *Arabidopsis* genome which might play redundant roles with *BAK1*. We overexpressed all 14 genes from the LRR-RLK II subfamily, to which the 5 SERKs belong, in the *bri1-5* background to evaluate their possible roles in BR signaling. Interestingly, only *SERK1*, *SERK2*, *BAK1*, and *BKK1* can suppress the defective phenotypes of *bri1-5* when overexpressed. None of the other 10 genes showed any *bri1-5* suppression phenotypes (Figure 1A, 1B).

We also overexpressed kinase-inactive mutants of all these 14 genes in *bri1-5*. Consistent with the *SERK* overexpression results, only *mSERK1*, *mSERK2*, *mBAK1*, *and mBKK1* give a dominant negative phenotype (Figure 1C). We therefore focused on generating single, double, tripe, and quadruple mutants of these four genes in order to estimate the genetic contribution of these four SERKs to BR signaling. Early studies indicated that *bak1 bkk1* double nulls are lethal due to failure of a BR-unrelated light-dependent cell death control pathway [36], [37], therefore we did not expect to observe that a multiple null mutant plant would recapitulate the *bri1* null mutant phenotype shown under light grown conditions, such as dark green, round and compact leaves, and extreme dwarfism [2]. Rather, we anticipated seeing that a multiple null mutant would express a de-etiolated phenotype resembling that of a *bri1* null mutant if SERKs play a key role in regulating BR signal transduction.

**Figure 7:**
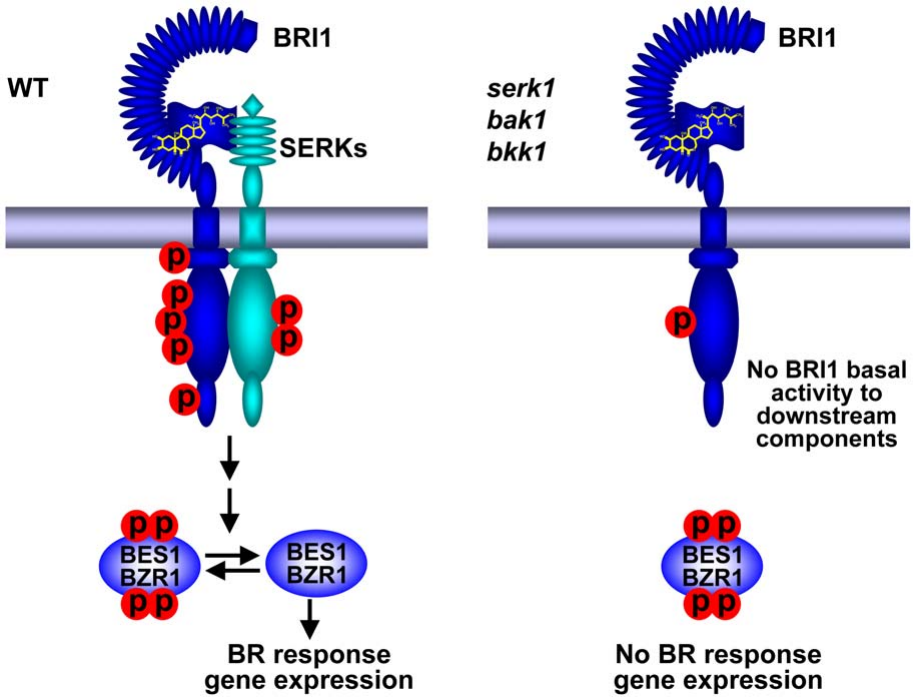
A current model showing SERKs are indispensable to BR signaling. Left: In wild-type plants, upon perception of BR by the receptor BRI1 and coreceptor BAK1 or its functionally redundant proteins, the phosphorylation levels of BRI1 respond to BR, triggering downstream BR signaling cascade, resulting the accumulation of unphosphorylated BES1, and ultimately leading to the expression of BR responsive genes. Right: In *serk1 bak1 bkk1* mutant plants, the phosphorylation levels of BRI1 remain at an almost undetectable basal level and do not alter regardless of elevated concentrations of BR. As a result, most of the BES1 protein exists as an inactive phosphorylated form, which cannot enter to the nuclei and is incapable to regulate the expression of BR responsive genes.

From our current results, we conclude that BAK1 and its homologues play an indispensable role in initiating BR signaling (Figure 7). This conclusion is mainly supported by the following key observations described in this study. First, the *serk1 bak1 bkk1* triple null mutant showed a characteristic de-etiolated phenotype similar to that of a null *bri1* mutant under dark grown conditions (Figure 3B, Figure S3B). Second, the triple null mutant is insensitive to BR according to the root growth inhibition analysis and the hypocotyl growth analysis (Figure 5A, 5B), *CPD*, *DWF4* feedback inhibition analysis (Figure 5C, 5D), and accumulation of unphosphorylated BES1 assay (Figure 5E, Figure S5). In the triple null mutant, no unphosphorylated forms of BES1 can be detected, indicating that the BR signaling pathway has been blocked, at least under a normal physiological setting; and the unphosphorylated levels of BES1 cannot be induced by exogenously applied BR, as shown in wild-type plants and other SERK single mutants (Figure 5E; Figure S5). Finally, overexpression of *BRI1* cannot alter the growth of the triple null mutant most likely because the phosphorylation of BRI1 is unresponsive to the fluctuation of the amount of internal biologically active BR (Figure 6). Together, these results suggest that the biological function of BRI1 in regulating plant growth is entirely dependent upon the action of SERKs.

SERK2 is apparently critical for the earlier development of embryos, as the development of the *serk1 serk2 bak1 bkk1* quadruple null mutant is completely arrested at an early embryo stage (unpublished data). No viable quadruple mutant seeds were ever recovered from these genetic analyses. Therefore, analysis of BR response in quadruple mutant was impracticable. Evidently, SERK2 plays no significant role at an early postembryonic seedling developmental stage; and the interaction between SERK2 and BRI1 is extremely weak compared to that of other SERK members and BRI1 (Figure S1) [10], [36], [46]. Exogenous application of BR cannot increase BRI1/SERK2 interactions (Figure S1). Under an unnatural condition, SERK2 may show BAK1-like biochemical properties. For example, seedlings treated with exogenously applied BR can drastically induce SERK2 phosphorylation (Figure S1); and overexpression of *SERK2* can partially suppress the defective phenotypes of *bri1-5* (Figure 1B). Our genetic and biochemical results, however, clearly indicate that SERK1, BAK1, and BKK1 are the major players for BR signaling at the *Arabidopsis* seedling stage.

It is worth noting that phenotypes of single, double, triple, and quadruple mutants of *SERK1*, *SERK2*, *BAK1*, and *BKK1* were discussed in a previous research article [50]. In that report, the authors claimed that *serk3 serk4* double mutant showed an early senescence but not a lethality phenotype. It was also reported that the *serk1 serk3 serk4* triple or *serk1 serk2 serk3 serk4* quadruple mutants did not show any enhanced phenotype over the *serk1 serk3* double mutant phenotype. The main cause for the discrepancy of our observations and the results from that report is that the mutant allele of *BAK1/SERK3* used in their genetic analyses, *serk3-1/bak1-3/SALK_ 034523*, is actually a leaky mutant. *bak1-3* contains a T-DNA insertion in the 4^th^ intron of the *BAK1* genomic DNA (Figure S6A). RT-PCR analysis and DNA sequence analysis indicated that *bak1-3* can express a reduced amount of wild-type *BAK1* mRNA in *bak1-3 bkk1-1* background, although the transcription level of *BAK1* in *bak1-3* single mutant is not detectable (Figure S6B). Consistent with these observations, *bak1-3* single mutant does exhibit a typical null *bak1* mutant phenotype which is indistinguishable from *bak1-4* (Figure S6C). *bak1-3 bkk1-1* double mutant, however, shows a much reduced early senescence phenotype than *bak1-4 bkk1-1* (Figure S6C). Therefore, *bak1-3* is not a true null mutant of *BAK1*, especially in the situation when *BKK1* is also knocked out (Figure S6B, [33]). In this study, we performed the genetic analysis by using two independent sets of confirmed transcriptional nulls (Figure S2B, S2C). Identical results were obtained from these two sets of mutant analyses (Figure 3A, 3B; Figure 5E; Figure S3A, S3B; Figure S5). In addition, the null *bri1-*like phenotype seen in the dark-grown *serk1 bak1 bkk1* triple mutant can be complemented with *BAK1* or *BKK1* driven by the *BAK1* promoter, but cannot be rescued by *SERK1* with its native promoter (Figure 4A, 4B). The later result is consistent with our previous discovery that the *bak1 bkk1* double null mutant is a seedling lethal mutant [36].

Our current observations suggest that SERKs play a critical role in the early events of BR signaling likely via a reciprocal and sequential phosphorylation model as proposed previously, with some modifications [20]. In wild-type plants, BR interacting with its receptor BRI1 in the extracellular domain triggers a conformational change within the cytoplasmic domain, leading to the basal activation of cytoplasmic BRI1 kinase, releasing of the BKI1 inhibitor, and inducing interaction between BRI1 and BAK1 or its homologues. The initially activated BRI1 evidently only can activate SERKs but not other downstream components such as BSKs. Activation of downstream components by BRI1 requires full activation of BRI1 by BAK1 and its functionally redundant homologues, most likely as a consequence of transphosphorylation. As a result, unphosphorylated forms of BES1 are accumulated which can directly regulate BR responsive gene expression (Figure 7). In a *serk1 bak1 bkk1* triple null mutant plant, on the other hand, BR signaling apparently is entirely blocked from BRI1 to BES1 due to lack of the involvement of BAK1 and its functionally redundant proteins (Figure 7). As a consequence, BES1 accumulates as the phosphorylated form, which is incapable of mediating the expression of BR-responsive genes. Therefore the triple null mutant plant exhibits a phenotype similar to a *bri1* null mutant. Both are the outcome of disrupted BR signaling. Although unphosphorylated BES1 level in both the *bri1* and *serk1 bak1 bkk1* triple null mutants showed almost an undetectable response to exogenously applied BR, the *bril* null mutant showed both phosphorylated and unphosphorylated BES1 (Figure 5E; Figure S5), suggesting a BR signal leakage in the *bri1* null mutant. This could be contributed by BRL1 and BRL3, both of which play partially redundant roles with BRI1 [9], [51]. In a previous report, it was hypothesized that without SERKs, BRI1 has basal kinase activity towards downstream components [20]. The hypothesis was based upon several observations from the *bak1 bkk1* double null mutant including that the de-etiolation of the *bak1 bkk1* is not as severe as the *bri1* null mutant; overexpression of *BRI1* can significantly increase hypocotyl growth of the *bak1 bkk1* double null mutant; and the phosphorylation levels of BRI1 can still respond to exogenous BR treatment. From this study, we now propose that without SERKs, BRI1 does not appear to have basal activities towards downstream components because the *serk1 bak1 bkk1* triple null shows a *bri1* null mutant phenotype; overexpression of BRI1 cannot increase the hypocotyl growth of the triple mutant; and in the triple null mutant the phosphorylation of BRI1 is completely unresponsive to BR treatment, hinting that BRI1 also cannot respond to internal BR levels. The BR signaling appears to be entirely blocked due to a blocked full BRI1 activation event by SERKs. In the future, analysis will be conducted to determine why phosphorylation of BRI1 cannot respond to BR in the absence of SERKs. The ultimate understanding of the roles of SERKs in BRI1-mediated signaling may rely on a combination of genetics and structural biology.

## Materials and Methods

### Plant materials, growth conditions, and crossing experiments

*Arabidopsis* accessions WS2 and Columbia-0 (Col-0) were grown at 22°C in a long-day condition (16 h of light and 8 h of dark) in a greenhouse except those for phenotypic analysis in the dark which were grown in half strength MS agar plates with 1% (w/v) sucrose. To create knock-out mutants used in this study, T-DNA insertion lines were obtained from The *Arabidopsis* Biological Resource center (ABRC) for *serk1-1* (SALK_044330), *serk1-8* (SALK_071511), *serk2-1* (SALK_058020), *serk2-2* (SAIL_119_G03), *bak1-3* (SALK_ 034523), *bak1-4* (SALK_116202), *bak1-6* (SAIL_513_A11), *bkk1-*1 (SALK_057955), *bkk1-2* (SALK_105409), *serk5-1* (SALK_089460), and *bri1-701* (SALK_003371).

Reverse transcription-polymerase chain reactions (RT-PCR) were performed to examine the expression of *BRI1* and *SERKs* in the used mutants. To amplify the full-length CDS and the flanking mRNA sequence of the T-DNA insertion site of *BRI1* and *SERKs*, primer pairs BRI1FL-F/BRI1FL-R, BRI1M-F/BRI1M-R, SERK1F-F/SERK1F-R, SERK1M-F/SERK1M-R, SERK2F-F/SERK2F-R, SERK2M-F/SERK2M-R, BAK1F/BAK1R, SERK3M-F/SERK3M-R, SERK4F-F/SERK4F-R, and SERK4M-F/SERK4M-R were used, respectively. *ACTIN2* was amplified as a control by primers RT-actin2F and RT-actin2R. All the used primers are listed in Table S1.

The T-DNA mutant plants were genotyped by PCR. Homozygous single knock-out lines were used to generate double knock-out mutants. Double knock-out mutants of *serk1 serk2*, and *bak1 bkk1* were obtained by segregating from corresponding mutant plants with homozygous insertion for one gene and heterozygous for the second gene. Triple knock-out mutants were obtained by crossing fertile pairs *serk1* ^+/−^ *serk2* and *serk2 bak1*, *serk1* ^+/−^ *serk2* and *serk2 bkk1*, *serk1 bak1* and *bak1* ^+/−^ *bkk1*, *serk2 bak1* and *bak1* ^+/−^ *bkk1*. The triple knock-out mutants were segregated from self-pollinated mutant plants *serk1* ^+/−^ *serk2 bak1*, *serk1* ^+/−^ *serk2 bkk1*, *serk1 bak1* ^+/−^ *bkk1* and *serk2 bak1* ^+/−^ *bkk1* which contain heterozygous insertion for one gene and homozygous insertions for two other genes.

### DNA cloning and plant transformation

Gateway technology was employed to clone all the coding sequences of *SERK* cDNA sequences for overexpression in *bri1-5* and complementation experiments in mutants as previously described [44] using the primers listed in Table S1. The amplified CDS sequences of *SERK* genes were introduced into the destination vector pB35GWF with the help of Gateway technology. Site-directed mutagenesis was carried out according to the manual of the QuickChange Site-directed Mutagenesis Kit (Stratagene, La Jolla, CA). Entry clones of cloned *SERK* genes were used as templates for chain extension with primers listed in Table S1. The mutated entry clones of *SERK* genes were used for *in vitro* DNA recombination with the destination vector pB35GWF to create expression constructs for *Arabidopsis* transformation.

To generate constructs for native promoter-driven expression of *SERKs*, promoter sequences of *SERKs* were PCR amplified from *Arabidopsis* genomic DNA and inserted into the *pBIB-BASTA-GWR-GFP* vector [44] before the gateway cassette by using the primers listed in Table S1. The resulting constructs were named as *pSERK1-GWR-GFP*, *pSERK2-GWR-GFP* and *pSERK3-GWR-GFP*, respectively. Then the cloned coding sequences of *SERK* genes were transferred into these destination vectors by *in vitro* DNA recombination to create expression constructs of *pS1::SERK1-GFP*, *pS2::SERK2-GFP*, *pS3::SERK3-GFP* and *pS3::SERK4-GFP*, respectively.

All the cloned sequences were confirmed by sequencing analysis and the expression constructs were transferred into appropriate *Arabidopsis* plants by the floral dip method [52].

### Root and hypocotyl growth analyses

Seeds were surface sterilized and placed on half strength MS plates with 0.8% (w/v) agar, 1% (w/v) sucrose and different concentrations of 24-epiBL (Sigma, St. Louis, MO). The plates were cold treated at 4°C for 2 days to ensure uniform germination. Seeds were considered to begin germination after the plates were kept at 22°C for 24 hr. The root length was measured seven days after germination in the light and the hypocotyl length was measured five days after germination in the dark.

### Membrane protein isolation, coimmunoprecipitation, and Western analysis

Seven-day-old seedlings of transgenic plants with *35S::BRI1-GFP* and 11-day-old liquid-cultured seedlings of transgenic plants harboring *35S::SERK2-GFP* and *35S::BRI1-FLAG* were treated with or without 24-epiBL for 90 min, respectively, and ground to fine powder in liquid N2 [17]. Membrane protein isolation was performed as previously described [10]. BRI1-FLAG was immunoprecipitated from solubilized membrane protein with agarose-linked α-FLAG antibody (Sigma, St. Louis, MO). SERK2-GFP and BRI1-GFP were immunoprecipitated with α-GFP antibody (Invitrogen, Carlsbad, CA) and protein G beads (Roche, Indianapolis, IN). The immunoprecipitated proteins were separated on 7.5% SDS polyacrylamide gel for Western analyses with α-GFP, α-FLAG, α-phosphothreonine antibodies as previously described [10]. *Arabidopsis* seedlings grown on half strength MS plates were harvested and treated with or without 1 µM 24-epiBL for 4 hr and the total protein was extracted and separated on 12% SDS polyacrylamide gel to detect the phosphorylation status of BES1 with an anti-BES1 antibody. Horseradish peroxidase-linked anti-rabbit or anti-mouse antibodies were used as secondary antibodies and the signal was detected by Western Lightning Chemiluminescence Reagent Plus (Perkin-Elmer, Waltham, MA).

## Supporting Information

Figure S1 SERK2 shows a basal level of interaction with BRI1. Co-immunoprecipitation result indicates that SERK2 can interact with BRI1 at a minimal level. The interaction cannot be enhanced by the exogenously applied BR. The phosphorylation of SERK2, on the other hand, is elevated to BR treatment.
(TIF)

Figure S2 *BRI1* and *SERK* mutants used in these studies do not express full-length mRNA. A. Expression of *BRI1* in *bri1-701* plants. RT-PCR reactions were performed to detect the full-length CDS (upper) and the mRNA sequence flanking the T-DNA insertion site (middle) in the wild type and the *bir1-701* mutant. The primer pairs used are indicated at the right. *ACTIN2* was amplified as a control (Lower). B, C. Expression of *SERKs* in *serk* mutant plants. The full-length CDS sequences and the mRNA sequences flanking T-DNA insertion sites were amplified with primer pairs listed in Table S1. The mutants are indicated on the top.
(TIF)

Figure S3 Representative loss-of-function mutant phenotypes of the mutants generated by an independent set of T-DNA insertion null mutants of *SERKs*. A. Representative loss-of-function phenotypes of 28-day-old *SERK* mutants in the light. Only *bak1-6* shows weak *bri1*-like phenotypes among the single knock-out mutants with smaller rosette size. The double knock-out mutant *serk1-1 bak1-6* shows similar phenotypes as the *bri1* weak allele *bri1-301*, and *bak1-6 bkk1-2* shows a seedling-lethality phenotype at the early developmental stage. The triple knock-out mutant *serk1-1 bak1-6 bkk1-2* shows similar seedling lethality phenotypes as the *bak1-6 bkk1-2* mutant plants. B. Representative loss-of-function phenotypes of 5-day-old *SERK* mutants in the dark. *bri1-701* shows a typical null *bri1* phenotype in the dark with opened cotyledons, shortened and swollen hypocotyls. Double null mutant *serk1-1 bak1-6* shows a similar phenotype to the *bri1-701*, with longer hypocotyls. The triple knock-out mutant *serk1-1 bak1-6 bkk1-2* shows similar phenotypes to the *bri1-701*, with completely opened cotyledons, shortened and swollen hypocotyls. C. Measurements of the dark-grown seedlings shown in B. Error bars represent SD.
(TIF)

Figure S4 The BR signaling pathway is not affected in the constitutive photomorphogenesis mutant *cop1*. A. Constitutive photomorphogenesis of 5-day-old *cop1* grown in the dark. B. *cop1* mutant plants are sensitive to exogenous BR treatment. The root length was measured for seven-day-old wild-type and mutant plants grown on 1/2 MS plates with 0 nM, 1 nM, 10 nM, 100 nM and 1000 nM of 24-epiBL, respectively. Error bars represent SD. C. BES1 phosphorylation level is responsive to exogenous BR treatment in *cop1* mutant plants similar to that in wild-type plants. Seven-day-old seedlings of wild-type and mutants grown in the light were treated with (+) or without (−) 1 µM 24-epiBL for 4 h. Total proteins were analyzed by Western hybridization with a specific anti-BES1 antibody. BES1 response upon BR treatment in the *cop1* mutants is similar to that of wild-type plants. Coomassie blue staining shows each pair of samples were equally loaded (Lower panel).
(TIF)

Figure S5 BES1 phosphorylation levels are not responsive to BR in the triple mutant *serk1-1 bak1-6 bkk1-2* generated by the 2^nd^ independent set of T-DNA insertion lines. A. Seven-day-old seedlings of wild-type and mutants grown in the light were treated with 0 or 1 µM 24-epiBL for 4 h. Total proteins were analyzed by Western hybridization with a specific anti-BES1 antibody. BES1 response upon BR treatment in the triple mutant *serk1-1 bak1-6 bkk1-2* is blocked. B. Coomassie blue staining of PAGE-separated proteins to show equally loaded proteins between each pair of treated and untreated samples. BL, BR treatment. −, without BR treatment; +, with BR treatment. BES1-P, phosphorylated BES1; BES1, unphosphorylated BES1.
(TIF)

Figure S6 *bak1-3* is a leaky T-DNA insertion mutant in *bak1-3 bkk1-1* background, whereas *bak1-4* is likely a null mutant. A. T-DNA insertion sites for *bak1-3* and *bak1-4*. Filled boxes represent exons, lines between boxes represent introns. The positions of the used primers are shown with arrows and the sequences are listed in Table S1. F: BAK1F; R: BAK1R; F1: BAK1F1; R1: BAK1R1; F2: BAK1F2; R2: BAK1R2. B. RT-PCR analyses indicated that *bak1-3* is a leaky mutant in *bkk1-1* background. In *bak1-3 bkk1-1* double but not in *bak1-3* single mutant background, wild-type *BAK1* cDNA can still be detected by RT-PCR. But there is no wild-type like full-length *BAK1* cDNA can be detected by RT-PCR in *bak1-4* single or *bak1-4 bkk1-1* double mutants. The used primer pairs are shown at the right. C. Phenotypes of 19-day-old wild-type, *bak1-3*, *bak1-4*, *bak1-3 bkk1-1*, and *bak1-4 bkk1-1*. *bak1-3* single mutant shows phenotypic defects similar to *bak1-4*; whereas *bak1-3 bkk1-1* double mutant shows a much milder phenotype than *bak1-4 bkk1-1*.
(TIF)

Table S1 Primers used for gene cloning, mutagenesis, real-time RT–PCR, and RT–PCR.
(DOC)
