# Supplementary figures and images for: Genetic Evidence for an Indispensable Role of Somatic Embryogenesis Receptor Kinases in Brassinosteroid Signaling
Source: PLoS Genet. 2012 Jan 12;8(1):e1002452. doi: 10.1371/journal.pgen.1002452 (PMC3257278; doi:10.1371/journal.pgen.1002452)

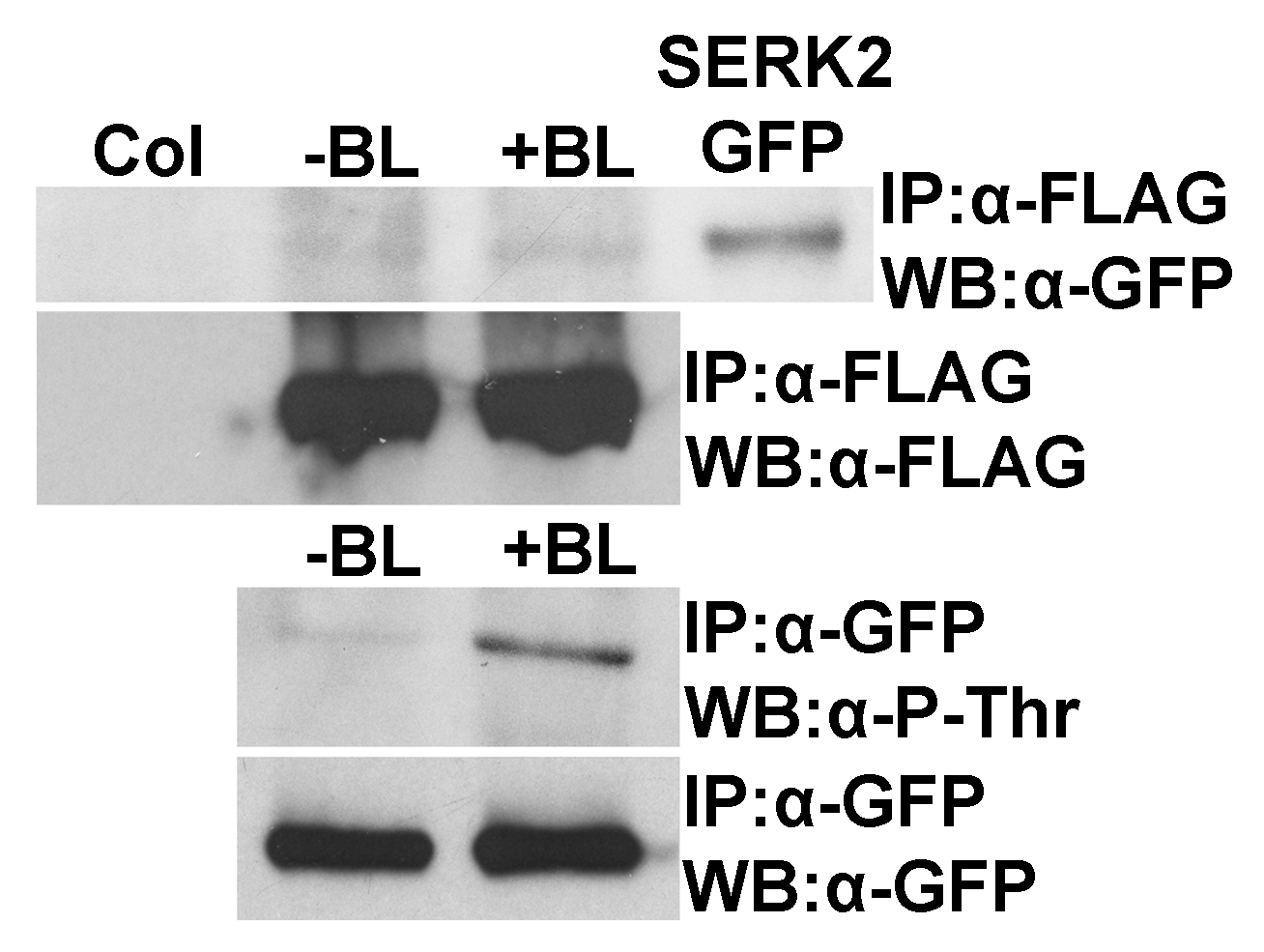

Supplement: Figure S1 — SERK2 shows a basal level of interaction with BRI1. Co-immunoprecipitation result indicates that SERK2 can interact with BRI1 at a minimal level. The interaction cannot be enhanced by the exogenously applied BR. The phosphorylation of SERK2, on the other hand, is elevated to BR treatment. (TIF) [file pgen.1002452.s001.tif]

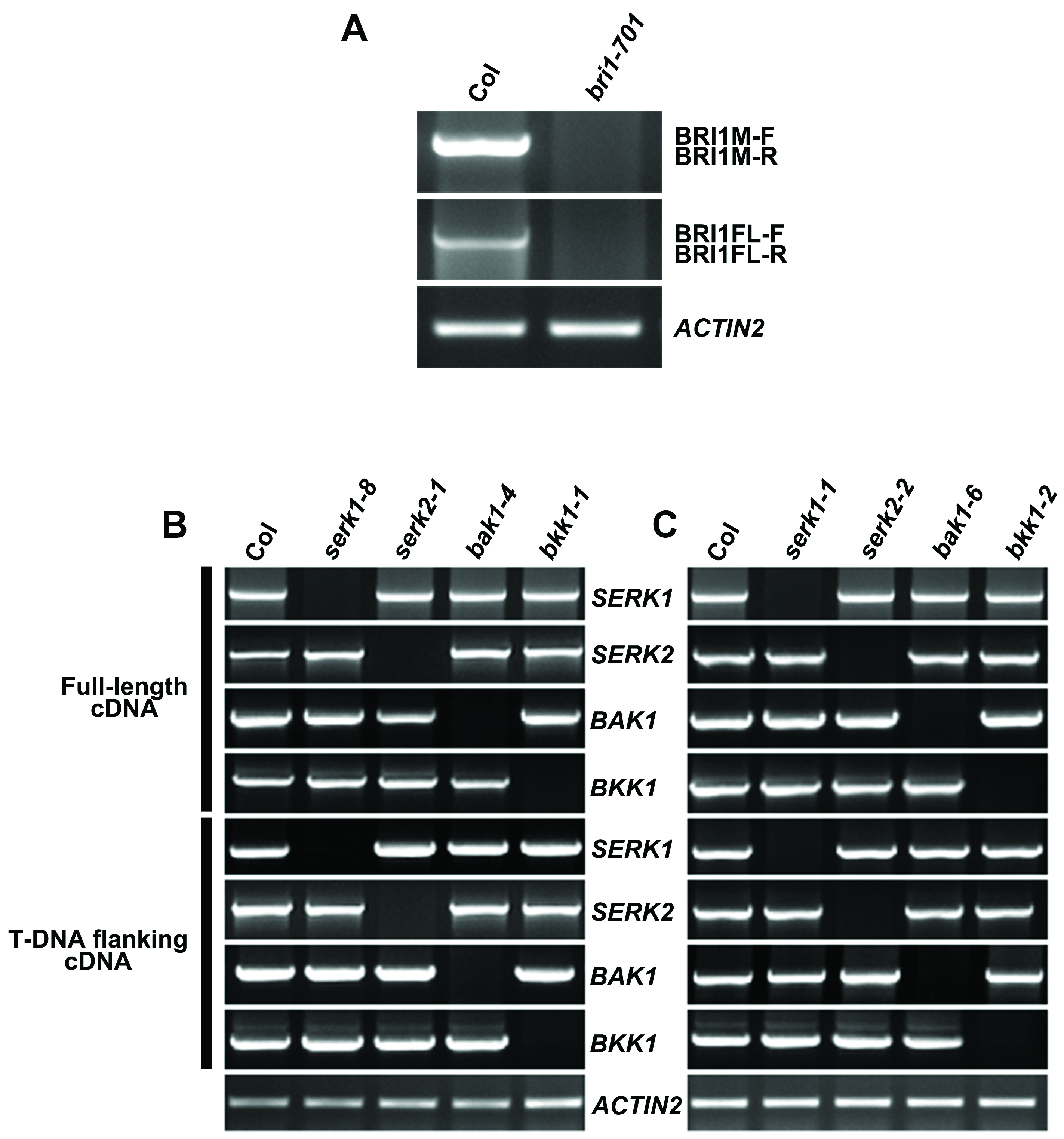

Supplement: Figure S2 — BRI1 and SERK mutants used in these studies do not express full-length mRNA. A. Expression of BRI1 in bri1-701 plants. RT-PCR reactions were performed to detect the full-length CDS (upper) and the mRNA sequence flanking the T-DNA insertion site (middle) in the wild type and the bir1-701 mutant. The primer pairs used are indicated at the right. ACTIN2 was amplified as a control (Lower). B, C. Expression of SERKs in serk mutant plants. The full-length CDS sequences and the mRNA sequences flanking T-DNA insertion sites were amplified with primer pairs listed in Table S1. The mutants are indicated on the top. (TIF) [file pgen.1002452.s002.tif]

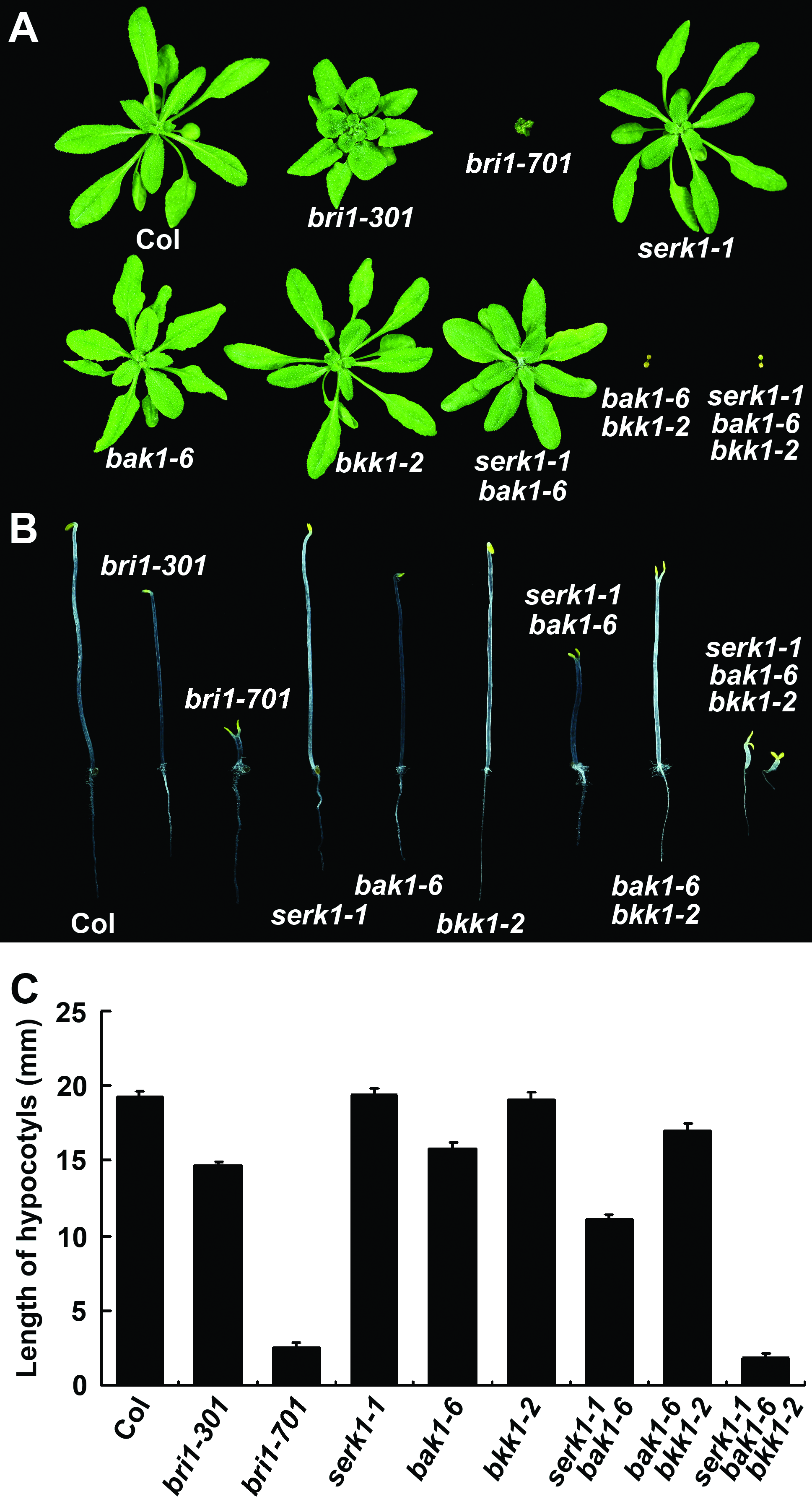

Supplement: Figure S3 — Representative loss-of-function mutant phenotypes of the mutants generated by an independent set of T-DNA insertion null mutants of SERKs. A. Representative loss-of-function phenotypes of 28-day-old SERK mutants in the light. Only bak1-6 shows weak bri1-like phenotypes among the single knock-out mutants with smaller rosette size. The double knock-out mutant serk1-1 bak1-6 shows similar phenotypes as the bri1 weak allele bri1-301, and bak1-6 bkk1-2 shows a seedling-lethality phenotype at the early developmental stage. The triple knock-out mutant serk1-1 bak1-6 bkk1-2 shows similar seedling lethality phenotypes as the bak1-6 bkk1-2 mutant plants. B. Representative loss-of-function phenotypes of 5-day-old SERK mutants in the dark. bri1-701 shows a typical null bri1 phenotype in the dark with opened cotyledons, shortened and swollen hypocotyls. Double null mutant serk1-1 bak1-6 shows a similar phenotype to the bri1-701, with longer hypocotyls. The triple knock-out mutant serk1-1 bak1-6 bkk1-2 shows similar phenotypes to the bri1-701, with completely opened cotyledons, shortened and swollen hypocotyls. C. Measurements of the dark-grown seedlings shown in B. Error bars represent SD. (TIF) [file pgen.1002452.s003.tif]

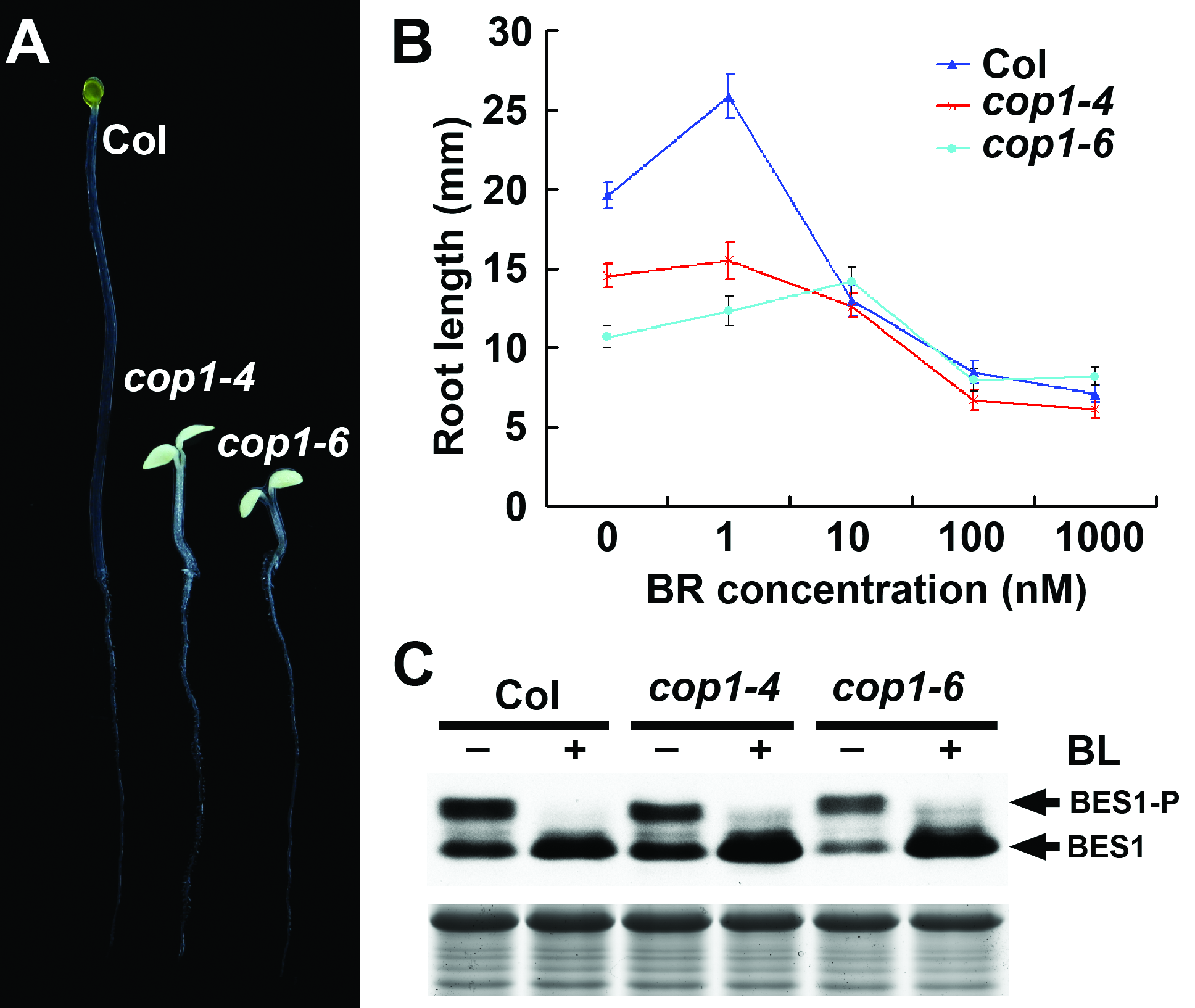

Supplement: Figure S4 — The BR signaling pathway is not affected in the constitutive photomorphogenesis mutant cop1. A. Constitutive photomorphogenesis of 5-day-old cop1 grown in the dark. B. cop1 mutant plants are sensitive to exogenous BR treatment. The root length was measured for seven-day-old wild-type and mutant plants grown on 1/2 MS plates with 0 nM, 1 nM, 10 nM, 100 nM and 1000 nM of 24-epiBL, respectively. Error bars represent SD. C. BES1 phosphorylation level is responsive to exogenous BR treatment in cop1 mutant plants similar to that in wild-type plants. Seven-day-old seedlings of wild-type and mutants grown in the light were treated with (+) or without (−) 1 µM 24-epiBL for 4 h. Total proteins were analyzed by Western hybridization with a specific anti-BES1 antibody. BES1 response upon BR treatment in the cop1 mutants is similar to that of wild-type plants. Coomassie blue staining shows each pair of samples were equally loaded (Lower panel). (TIF) [file pgen.1002452.s004.tif]

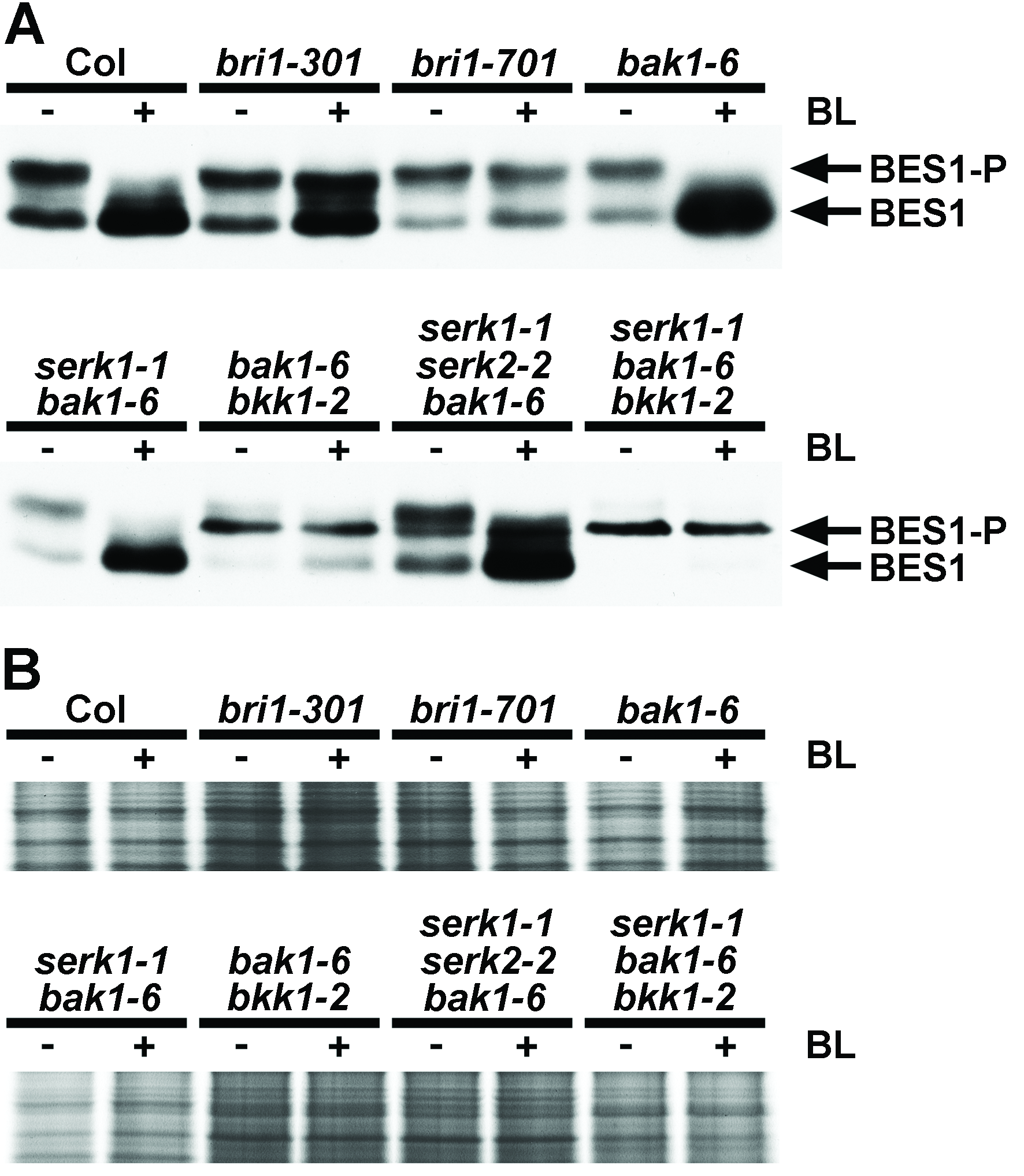

Supplement: Figure S5 — BES1 phosphorylation levels are not responsive to BR in the triple mutant serk1-1 bak1-6 bkk1-2 generated by the 2nd independent set of T-DNA insertion lines. A. Seven-day-old seedlings of wild-type and mutants grown in the light were treated with 0 or 1 µM 24-epiBL for 4 h. Total proteins were analyzed by Western hybridization with a specific anti-BES1 antibody. BES1 response upon BR treatment in the triple mutant serk1-1 bak1-6 bkk1-2 is blocked. B. Coomassie blue staining of PAGE-separated proteins to show equally loaded proteins between each pair of treated and untreated samples. BL, BR treatment. −, without BR treatment; +, with BR treatment. BES1-P, phosphorylated BES1; BES1, unphosphorylated BES1. (TIF) [file pgen.1002452.s005.tif]

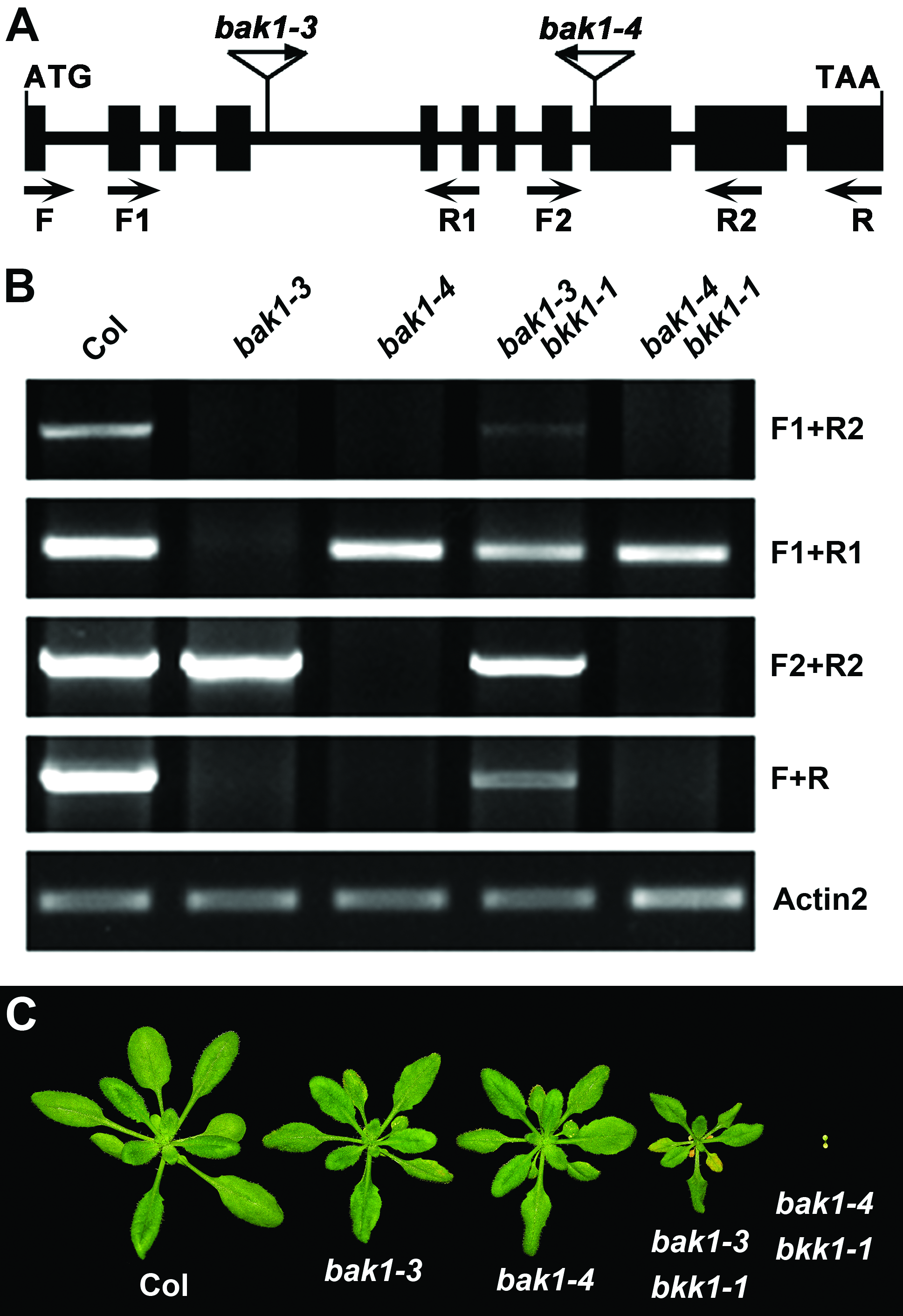

Supplement: Figure S6 — bak1-3 is a leaky T-DNA insertion mutant in bak1-3 bkk1-1 background, whereas bak1-4 is likely a null mutant. A. T-DNA insertion sites for bak1-3 and bak1-4. Filled boxes represent exons, lines between boxes represent introns. The positions of the used primers are shown with arrows and the sequences are listed in Table S1. F: BAK1F; R: BAK1R; F1: BAK1F1; R1: BAK1R1; F2: BAK1F2; R2: BAK1R2. B. RT-PCR analyses indicated that bak1-3 is a leaky mutant in bkk1-1 background. In bak1-3 bkk1-1 double but not in bak1-3 single mutant background, wild-type BAK1 cDNA can still be detected by RT-PCR. But there is no wild-type like full-length BAK1 cDNA can be detected by RT-PCR in bak1-4 single or bak1-4 bkk1-1 double mutants. The used primer pairs are shown at the right. C. Phenotypes of 19-day-old wild-type, bak1-3, bak1-4, bak1-3 bkk1-1, and bak1-4 bkk1-1. bak1-3 single mutant shows phenotypic defects similar to bak1-4; whereas bak1-3 bkk1-1 double mutant shows a much milder phenotype than bak1-4 bkk1-1. (TIF) [file pgen.1002452.s006.tif]
