# Supplementary material for: Genetic Evidence for an Indispensable Role of Somatic Embryogenesis Receptor Kinases in Brassinosteroid Signaling
Source: PLoS Genet. 2012 Jan 12;8(1):e1002452. doi: 10.1371/journal.pgen.1002452 (PMC3257278; doi:10.1371/journal.pgen.1002452)
Supplement: Table S1 — Primers used for gene cloning, mutagenesis, real-time RT–PCR, and RT–PCR. (DOC) [file pgen.1002452.s007.doc]

**Table S1** Primers used for gene cloning, mutagenesis, real-time RT-PCR and RT-PCR

| **Primer name** | **Sequence** |
| --- | --- |
| **LRR-RLK II cloning** | |
| SERK1PB1 | AAAAAGCAGGCTTCATGGAGTCGAGTTATGTGGTGTTTA |
| SERK1PB2 | AGAAAGCTGGGTCCCTTGGACCAGATAACTCAACG |
| SERK2PB1 | AAAAAGCAGGCTTCATGGGGAGAAAAAAGTTTGAAGCT |
| SERK2PB2 | AGAAAGCTGGGTCTCTTGGACCAGACAACTCCATAGCA |
| SERK3PB1 | AAAAAGCAGGCTTCATGGAACGAAGATTAATGATCCCT |
| SERK3PB2 | AGAAAGCTGGGTCTCTTGGACCCGAGGGGTATTCGT |
| SERK4PB1 | AAAAAGCAGGCTTCATGACAAGTTCAAAAATGGAACA |
| SERK4PB2 | AGAAAGCTGGGTCTCTTGGACCCGAGGGGTAATCGT |
| SERK5PB1 | AAAAAGCAGGCTTCATGGAACATGGATCATCCCGTG |
| SERK5PB2 | AGAAAGCTGGGTCTCTTGGCCCCGAGGGGTAATCGT |
| 1g60800PB1 | AAAAAGCAGGCTTCATGGAAGGTGTGAGATTTGTGGTGT |
| 1g60800PB2 | AGAAAGCTGGGTCTCGAGGACCCGAGAGCTCAATGGCT |
| 2g23950PB1 | AAAAAGCAGGCTTCATGGTGGTGATGAAGTTAATAACAAT |
| 2g23950PB2 | AGAAAGCTGGGTCCCTTGGACCAGATAGTTCCATG |
| 3g25560PB1 | AAAAAGCAGGCTTCATGTTGCAAGGAAGAAGAGAAGCA |
| 3g25560PB2 | AGAAAGCTGGGTCTCTTGGACCTGATAACTCCATGGCT |
| 4g30520PB1 | AAAAAGCAGGCTTCATGGTAGTAGTAACAAAGAAGACCATG |
| 4g30520PB2 | AGAAAGCTGGGTCTCTTGGACCGGATAGTTCCATG |
| 5g10290PB1 | AAAAAGCAGGCTTCATGAGAATGTTCAGCTTGCAGAAGA |
| 5g10290PB2 | AGAAAGCTGGGTCTCTTCCACCAGATAATTCAATGGCA |
| 5g16000PB1 | AAAAAGCAGGCTTCATGGAGAGTACTATTGTTATGATGATGA |
| 5g16000PB2 | AGAAAGCTGGGTCTCTAGGACCAGAGAGCTCCATTG |
| 5g45780PB1 | AAAAAGCAGGCTTCATGGAGATTTCTTTGATGAAGT |
| 5g45780PB2 | AGAAAGCTGGGTCTCGTGGTCCAGAGAGCTCAATG |
| 5g63710PB1 | AAAAAGCAGGCTTCATGGCTCACTCGGGGAACGGTGA |
| 5g63710PB2 | AGAAAGCTGGGTCTCTTGCTGTCGATAATCGGATAGAT |
| 5g65240PB1 | AAAAAGCAGGCTTCATGGCTCTGCTTATTATCACTGCCTTA |
| 5g65240PB2 | AGAAAGCTGGGTCTCTTCCACCAGATAATTCAATAGCA |
| **LRR-RLK II site-directed mutagenesis** | |
| SERK1K330E(+) | ACTCTTGTTGCTGTCGAGAGACTGAAGGAAG |
| SERK1K330E(-) | CTTCCTTCAGTCTCTCGACAGCAACAAGAGT |
| SERK2K333E(+) | ACACTTGTTGCAGTCGAACGGCTTAAAGAAG |
| SERK2K333E(-) | CTTCTTTAAGCCGTTCGACTGCAACAAGTGT |
| BAK1K317E(+) | ACTTTAGTGGCCGTTGAAAGGCTAAAAGAGG |
| BAK1K317E(-) | CCTCTTTTAGCCTTTCAACGGCCACTAAAGT |
| BKK1K322E(+) | AATCTAGTGGCTGTCGAAAGGCTAAAAGAAG |
| BKK1K322E(-) | CTTCTTTTAGCCTTTCGACAGCCACTAGATT |
| 1g60800K329E(+) | ACTTTGGTGGCTGTCGAACGTCTCAAGGACT |
| 1g60800K329E(-) | AGTCCTTGAGACGTTCGACAGCCACCAAAGT |
| 2g23950K327E(+) | ACAGTGGTTGCAGTGGAACGATTGAAAGATG |
| 2g23950K327E(-) | CATCTTTCAATCGTTCCACTGCAACCACTGT |
| 3g25560K340E(+) | AGTATCATCGCGGTGGAGAGATTAAAGGATA |
| 3g25560K340E(-) | TATCCTTTAATCTCTCCACCGCGATGATACT |
| 4g30520K330E(+) | ACAATGGTGGCAGTGGAACGGTTGAAGGATA |
| 4g30520K330E(-) | TATCCTTCAACCGTTCCACTGCCACCATTGT |
| 5g10290K318E(+) | ACCAAAGTTGCTGTGGAGAGATTGACGGATT |
| 5g10290K318E(-) | AATCCGTCAATCTCTCCACAGCAACTTTGGT |
| 5g16000K340E(+) | ACAGTGGTTGCAGTGGAAAGGCTTAAAGATG |
| 5g16000K340E(-) | CATCTTTAAGCCTTTCCACTGCAACCACTGT |
| 5g45780K328E(+) | ACTGTGGTGGCAGTTGAAAGATTGAAAGATC |
| 5g45780K328E(-) | GATCTTTCAATCTTTCAACTGCCACCACAGT |
| 5g63710K317E(+) | ACAAAAGTTGCAGTGGAACGCCTTGCGGATT |
| 5g63710K317E(-) | AATCCGCAAGGCGTTCCACTGCAACTTTTGT |
| 5g65240K322E(+) | ACCAAAGTCGCTGTAGAAAGATTGACTGATT |
| 5g65240K322E(-) | AATCAGTCAATCTTTCTACAGCGACTTTGGT |
| **Promoter cloning** | |
| SERK1HindIIIF | ACAAAGCTTCTCTTTCATAACAAGGTAGCTCTT |
| SERK1Kpn1R | ACAGGTACCTTCAAACAACAATGCTAAATTTCG |
| SERK2HindIIIF | ACAAAGCTTACAATCTCTGCACCTTTCTTATAT |
| SERK2XbaIR | ACATCTAGATTACCAAAAAAAAGCAAATTTCTC |
| BAK1PromFXba | TCTTCTAGAGTGGGACTTTTAGATGGTTGGAGA |
| BAK1PromRKpn | TAAGGTACCTTTATCCTCAAGAGATTAAAAACAAAC |
| ***CPD*, *DWF4* real-time RT-PCR** | |
| CPDF | GAGACGCTACGAGTGGCTAA |
| CPDR | GCATCTTTGAAGTGGTTTGGG |
| DWF4F | CCACAACACTCGGTGACTTC |
| DWF4R | CAGCTGATACGATCGTTGGTT |
| Actin2F | TGTGCCAATCTACGAGGGTTT |
| Actin2R | TTTCCCGCTCTGCTGTTGT |
| ***SERKs*, *BRI1* RT-PCR** | |
| BAK1F | ATGGAACGAAGATTAATGATCCC |
| BAK1R | TTATCTTGGACCCGAGGGGTATT |
| BAK1F1 | CACTATTGTCGCTATTGCAAG |
| BAK1R1 | GAACTTGTAGCGTCAGGACAG |
| BAK1F2 | GTTGACTCCCCTTCCTGCAT |
| BAK1R2 | CATCATCATCATTCGCGAGG |
| SERK1F-F | ATGGAGTCGAGTTATGTGGTG |
| SERK1F-R | CCTTGGACCAGATAACTCAAC |
| SERK1M-F | GGTATAACTGGAGCAATAGCTG |
| SERK1M-R | CTCAACTTTTTGCCATTCGTCC |
| SERK2F-F | GGGGAGAAAAAAGTTTGAAGC |
| SERK2F-R | GACCAGACAACTCCATAGCAT |
| SERK2M-F | GTGATGCACTGCACAGTTTG |
| SERK2M-R | GATTTCTGTGAACGGCCATG |
| SERK3M-F | GTGATGCTCTAAGTGCACTG |
| SERK3M-R | CTGTCTCTTTGGCCAATCAAG |
| SERK4F-F | GTCTTCGTCAACGGCTGAATC |
| SERK4F-R | CCCACAAGAAAAGACCCTTTC |
| SERK4M-F | CCTGAGAATAAAGTTACTCGTG |
| SERK4M-R | GTTGTCACATGGGAGTCATT |
| BRI1FL-F | ATGAAGACTTTTTCAAGCTTC |
| BRI1FL-R | TAATTTTCCTTCAGGAACTTC |
| BRI1M-F | GCTTCAAAGACGTTCTTCCTG |
| BRI1M-R | GGATCTGATTCCTTGAAACTC |
| RT-actin2F | CAGTGGTCGTACAACCGGTATTG |
| RT-actin2R | TGCTGTGATTTCTTTGCTCATACG |
